# Characterization of Endophytic Bacteria Isolated from *Typha latifolia* and Their Effect in Plants Exposed to Either Pb or Cd

**DOI:** 10.3390/plants12030498

**Published:** 2023-01-21

**Authors:** Jesús Rubio-Santiago, Alejandro Hernández-Morales, Gisela Adelina Rolón-Cárdenas, Jackeline Lizzeta Arvizu-Gómez, Ruth Elena Soria-Guerra, Candy Carranza-Álvarez, Jocabed Eunice Rubio-Salazar, Stephanie Rosales-Loredo, Juan Ramiro Pacheco-Aguilar, José Roberto Macías-Pérez, Liseth Rubí Aldaba-Muruato, Juan Vázquez-Martínez

**Affiliations:** 1 Facultad de Ciencias Químicas, Universidad Autónoma de San Luis Potosí, San Luis Potosí 78210, Mexico; 2 Facultad de Estudios Profesionales Zona Huasteca, Universidad Autónoma de San Luis Potosí, San Luis Potosi 79060, Mexico; 3 Secretaría de Investigación y Posgrado, Centro Nayarita de Innovación y Transferencia de Tecnología (CENITT), Universidad Autónoma de Nayarit, Tepic 63173, Mexico; 4 Departamento de Diagnóstico Molecular, Universidad de Monterrey, San Pedro Garza García 66238, Mexico; 5 Facultad de Química, Universidad Autónoma de Querétaro, Santiago de Querétaro 63173, Mexico; 6 Departamento de Ingeniería Química y Bioquímica; Tecnológico Nacional de México Campus Irapuato, Guanajuato 36821, Mexico

**Keywords:** phytoremediation, *Typha latifolia*, plant-growth-promoting rhizobacteria

## Abstract

Plant-associated bacteria in heavy-metal-contaminated environments could be a biotechnological tool to improve plant growth. The present work aimed to isolate lead- and cadmium-tolerant endophytic bacteria from the roots of *Typha latifolia* growing in a site contaminated with these heavy metals. Endophytic bacteria were characterized according to Pb and Cd tolerance, plant-growth-promoting rhizobacteria activities, and their effect on *T. latifolia* seedlings exposed and non-exposed to Pb and Cd. Pb-tolerant isolates were identified as *Pseudomonas azotoformans* JEP3, *P. fluorescens* JEP8, and *P. gessardii* JEP33, while Cd-tolerant bacteria were identified as *P. veronii* JEC8, JEC9, and JEC11. They all exert biochemical activities, including indole acetic acid synthesis, siderophore production, and phosphate solubilization. Plant–bacteria interaction assays showed that *P. azotoformans* JEP3, *P. fluorescens* JEP8, *P. gessardii* JEP33, and *P. veronii* JEC8, JEC9, JEC11 promote the growth of *T. latifolia* seedlings by increasing the root and shoot length, while in plants exposed to either 5 mg/L of Pb or 10 mg/L of Cd, all bacterial isolates increased the shoot length and the number of roots per plant, suggesting that they are plant-growth-promoting rhizobacteria that could contribute to *T. latifolia* adaptation to the heavy metal polluted site.

## 1. Introduction

Lead (Pb) and Cadmium (Cd) are two heavy metals used in many industrial processes, so their environmental levels have increased considerably [1]. They have no beneficial role in biological systems and are highly toxic for living organisms even at low concentrations [2]. Pb and Cd pollution is a major concern worldwide because they are stable, persistent, and cannot be biodegraded. Therefore, they accumulate in different crops and incorporate into the food chain, causing chronic and acute disorders in humans [3]. Physicochemical methods have been developed to reduce Pb and Cd concentrations in the environment and thus avoid harmful effects on human health and ecosystems. Despite their effectiveness, most are expensive, inefficient, and non-eco-friendly [4]. Phytoremediation is an environmentally friendly strategy that uses plant species to remove Pb and Cd from soil and water [4]. For instance, *Paspalum fasciculatum* [5], *Helianthus annuus* [6], and *Panicum virgatum* [7] have been used to remove Pb and Cd from contaminated soils. 

The *Typha* genus is another of the most important in phytoremediation. It includes plant species commonly known as cattail, and is used to remove heavy metals from water, soil, and sediments in natural and artificial wetlands [8]. *T. angustifolia*, *T. domingensis*, and *T. latifolia* have been used in phytoremediation due to their fast growth, large biomass, and ability to accumulate Pb, Cd, and other heavy metals mainly in their roots [9]. 

Heavy-metal-tolerant plants have been shown to interact with plant-growth-promoting rhizobacteria (PGPR), which contribute to their growth and adaptation to rhizosphere conditions through activities such as phosphate solubilization, siderophore production, indole acetic acid synthesis (IAA), and 1-aminocyclopropane 1-carboxylic acid (ACC) deaminase activity [10]. 

The study of plant–bacteria interactions for heavy metals remediation has gained importance recently. It has been demonstrated that the roots of *Spartina maritima*, *S. densiflora*, and *Sulla coronaria* that grow in heavy metal polluted environments are colonized by heavy-metal-tolerant PGPR, which increase the plants’ heavy metal tolerance and even improve phytoremediation [11,12,13]. 

Regarding PGPR associated with *Typha* species, it has been shown that the endosphere of *T. angustifolia* roots growing in wastewater wetland is colonized by bacteria belonging to the phylum *Proteobacteria*, which includes bacteria with the potential to improve plant growth and enhance phytoremediation of eutrophic water bodies [14]. Furthermore, the roots of *T. angustifolia* growing in a uranium mine tailing contaminated with iron are colonized by *Paenibacillus cookii* JGR8, *Pseudomonas jaduguda* JGR2, and *Bacillus megaterium* JGR9. Although these bacteria exhibit PGPR activities, including siderophore production, IAA synthesis, and phosphate-solubilizing capacity, only *P. cookii* JGR8 promotes the growth of its host, *T. angustifolia*, compared with non-inoculated plants [15]. Likewise, Saha, et al. [16] demonstrated that the root endosphere of *T. angustifolia* is colonized by *Staphylococcus*, *Bacillus, Planococcus*, *Kocuria*, *Micrococcus*, *Pseudomonas* sp., and *Sphingomonas* species. A consortium including these bacteria promotes the growth of *T. angustifolia* and rice through PGPR activities such as auxin synthesis, siderophore production, and nitrogen fixation [16]. 

Furthermore, *Pseudomonas gessardii* isolated from the *T. domingensis* rhizosphere has been used to acclimatize, promote the growth, and enhance Fe, Mn, Ni, Pb, and Cr uptake by the roots of four macrophytes, *Brachia mutica*, *T. domingensis*, *Phragmites australis*, and *Leptochala fusca*, in a constructed wetland to treat polluted river water [17]. 

Regarding *T. latifolia*, little is known about the bacterial populations associated with its roots and their contribution to the plants´ tolerance to heavy metal or their influence on improving phytoremediation [18,19,20]. Recently, Rolón-Cárdenas, et al. [21] demonstrated that four Cd-tolerant endophytic *Pseudomonas rhodesiae* strains, isolated from the roots of *T. latifolia*, exert PGPR activities and improve the growth of *Arabidopsis thaliana* seedlings exposed to 2.5 mg/L of Cd. No other heavy-metal-tolerant bacterial strains from *T. latifolia* have been described so far. Although bacteria belonging to the *Pseudomonas* genus have been isolated from the roots of *T. latifolia* growing in a constructed wetland to remove diclofenac and naproxen, their heavy metal tolerance remains unknown [22]. 

Therefore, in this study, endophytic bacteria were isolated from *T. latifolia* roots growing in a heavy-metal-contaminated site, with the aim of obtaining further insight into the bacteria that contribute to plant adaptation to a heavy-metal-contaminated environment. Bacteria were characterized based on Pb and Cd tolerance and PGPR activities, while the most tolerant bacterial strains were tested in plant–bacteria interaction assays using *T. latifolia* seedlings exposed to either Pb or Cd. 

## 2. Results

### 2.1. Pb and Cd Content in the Soil and Roots of T. latifolia

Previously, Rolón-Cárdenas et al. reported the cadmium content in the study site, being 5.65 ± 0.01 mg/Kg of Cd per dry weight of soil and 1.82 ± 0.04 mg/kg of Cd per dry weight of *T. latifolia* roots, with a bioconcentration factor (BCF) of 0.32, which indicated that *T. latifolia* takes up Cd from the soil and accumulates it in its roots [21]. 

The results of Pb quantitation showed that the soil contained 72.17 ± 0.84 mg/Kg of Pb per dry weight and the *T. latifolia* roots contained 12.28 ± 0.30 mg/Kg of Pb per dry weight, and a bioconcentration factor (BCF) of 0.17 for Pb. These results indicated that *T. latifolia* takes up Pb from the surrounding soil and accumulates it in its roots. 

### 2.2. Isolation of Pb- and Cd-Tolerant Endophytic Bacteria from T. latifolia Roots

**Figure 1:**
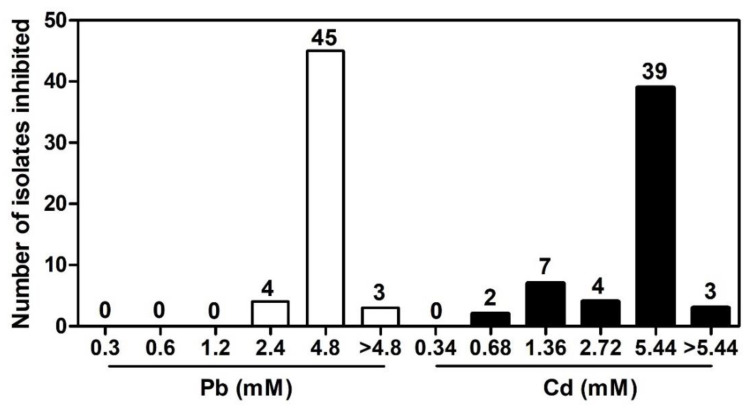
Minimum inhibitory concentrations (MIC) of Pb and Cd for bacterial isolates from *Typha latifolia* roots. The values above the bar show the number of isolates inhibited at each Pb or Cd concentration.

Endophytic bacteria were isolated in LB agar supplemented with either Pb 0.02 mM or Cd 0.04 mM, used as selection marker. A total of 107 Pb- and Cd-tolerant endophytic bacteria were isolated from *T. latifolia* roots, of which 52 and 55 isolates were obtained in LB media with Pb 0.02 mM and Cd 0.04 mM, respectively. MIC tests were performed to determine whether endophytic bacteria tolerate higher Pb and Cd concentrations than those used in preliminary isolation. The results showed that Pb-tolerant endophytic bacteria tolerate concentrations ranging from 0.3 to 4.8 mM of Pb; however 4.8 mM Pb inhibited 86% (45/52) of the isolates. Three isolates (JEP3, JEP8, and JEP33) presented an MIC for Pb > 4.8 mM (Figure 1). For Cd, we observed that endophytic bacteria tolerate concentrations ranging from 0.34 to 5.44 mM, and 5.44 mM of Cd was the concentration that inhibited 71% (39/55) of the isolates. Three isolates (JEC8, JEC9, and JEC11) showed the highest tolerance to Cd (MIC > 5.44 mM) (Figure 1). These results indicated that the roots of *T. latifolia* growing at the sampled site are colonized by Pb- and Cd-tolerant endophytic bacteria. The most tolerant isolates for Pb (JEP3, JEP8, and JEP33) and Cd (JEC8, JEC9, and JEC11) were selected for further studies. 

### 2.3. Identification of Bacterial Isolates

To identify the most Pb- and Cd-tolerant bacterial isolates (JEP3, JEP8, JEP33, JEC8, JEC9, and JEC11), we amplified, sequenced, and analyzed the 16S *rRNA*, *gyrA*, and *rpoD* genes. The results showed that sequences of six bacterial isolates had high similarity (>95%) with the *Pseudomonas* genus. Pb-tolerant endophytic bacteria, JEP3, JEP8, and JEP33, were identified as *P. azotoformans*, *P. fluorescens*, and *P. gessardii*, respectively. While all Cd-tolerant endophytic bacteria, JEC8, JEC9, and JEC11, were identified as *P. veronii* (Table 1). 

### 2.4. Antibiotic Susceptibility Test and Osmotic Tolerance

The results showed that the six bacterial isolates were resistant to multiple antibiotics with a MAR Index > 2.0. Bacterial isolates resisted the antibiotic classes of tetracyclines, quinolones, and aminoglycosides (Table 2). Additionally, *P. fluorescens* JEP8 and *P. veronii* JEC11 showed resistance to ceftriaxone, whereas *P. veronii* JEC8 showed resistance to chloramphenicol (Table 2). 

After the antibiotic susceptibility test, osmotic tolerance was evaluated. The results showed that the six bacterial strains grew in LB media supplemented with high NaCl, sucrose, and glycerol concentrations. The maximum tolerable concentration for all bacterial strains was 1.0 M of NaCl, 1.0 M of sucrose, and >2.0 M of glycerol, indicating the bacterial ability to tolerate osmotic stress. 

### 2.5. Screening for PGPR Abilities in Endophytic Bacterial Isolates

**Figure 2:**
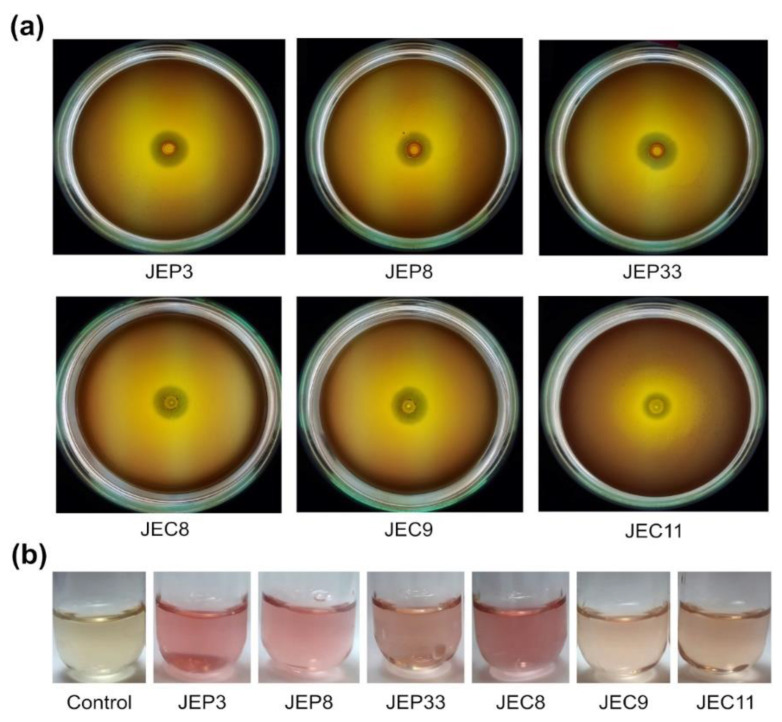
Plant-growth-promoting rhizobacteria (PGPR) traits of Pb- and Cd-tolerant bacterial isolates from *Typha latifolia* roots. (**a**) Phosphate solubilization activity of bacterial strains in NBRIP media after 15 days of incubation. A clear halo around the bacterial colony indicates phosphate solubilization. (**b**) Colorimetric determination of IAA and indole compounds in bacterial supernatants using Salkowski reaction. A pink color indicates positive reaction.

PGPR abilities were determined in the six Pb- and Cd-tolerant bacterial strains (JEP3, JEP8, JEP33, JEC8, JEC9, and JEC11). The six bacterial strains developed an orange halo around the colony in CAS media, indicating siderophore production in the iron deficiency medium (Table 3). The ACC deaminase activity was detected in DF media with ACC as a nitrogen (N) source. The results showed that except for JEP8, all bacterial strains grew in DF media with ACC, indicating that they metabolized ACC as an N source (Table 3). The phosphate-solubilizing ability of the strains were performed in NBRIP media with Ca(PO_3_)_2_ as a unique phosphate source. In NBRIP agar, the six bacterial strains formed a clear halo around the colony, indicating their capacity to solubilize phosphate from Ca(PO_3_)_2_ (Table 3, Figure 2a). Indole-related compound production was determined by a colorimetric method using Salkowsky’s reagent (Figure 2b). All Cd- and Pb-tolerant bacterial isolates produce indole-related compounds at 6.3–31.9 µg/mL. The IAA production by the bacterial strains was confirmed by GC-MS analysis of extracts of the supernatant of the LB medium with L-Trp. The GC-MS analysis showed that the six Cd- and Pb-tolerant bacteria strains could produce IAA from L-Trp in LB medium (Table 3). These results indicated that JEP3, JEP8, JEP33, JEC8, JEC9, and JEC11 have biochemical activities related to plant growth promotion, suggesting that they are PGPR. 

### 2.6. Effect of Bacterial Isolates in T. latifolia Seedlings

**Figure 3:**
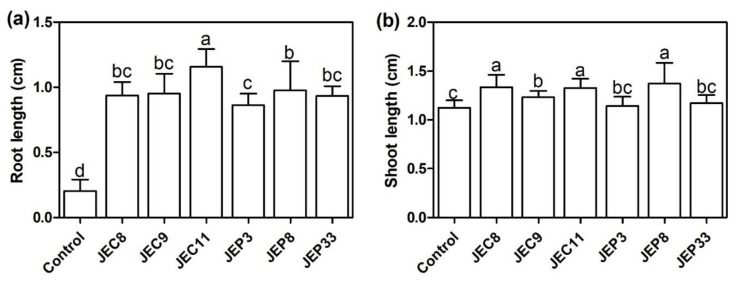
Effect of Pb- and Cd-tolerant bacterial strains on *T. latifolia*. *T. latifolia* seeds were mixed with bacterial suspensions and incubated in water-agar 0.7% (*w*/*v*) for 10 days. (**a**) Root length and (**b**) shoot length after incubation. Data represent the mean ± SD. Different letters indicate significant differences between treatments (*p* < 0.05).

To determine the role of endophytic bacteria in plants, the roots of *T. latifolia* seedlings were dipped in bacterial strains suspensions and sowed in MS medium. Results showed that all bacterial strains significantly increased the root length of seedlings compared with the control (*p* < 0.05) (Figure 3a). *P. veronii* JEC11 was the most effective bacterium, increasing the root length 6-fold, while *P. azotoformans* JEP3 was the least effective, increasing 4.5-fold compared with the control seedlings. On the other hand, only *P. veronii* JEC8, JEC9, and JEC11 and *P. fluorescens* JEP8 significantly increased the shoot length compared to the control (*p* < 0.05) (Figure 3b). These results suggested that endophytic bacteria isolated from *T. latifolia* roots promote the growth of the host plant. 

### 2.7. Effect of Bacterial Strains on T. latifolia Seedlings Exposed to Either Pb or Cd

Initially, the Pb effects were determined in *T. latifolia* seedlings growing in MS medium with this metal in doses ranging from 5 to 30 mg/L of Pb for ten days. Results showed that Pb significantly inhibited root and shoot elongation in concentrations from 5 mg/L and higher compared to non-exposed seedlings (*p* < 0.05) (Supplementary Material Figure S1a,b). On the other hand, doses ranging from 10 to 50 mg/L of Cd were tested in *T. latifolia* seedlings, showing that Cd significantly inhibited root elongation in concentrations from 10 mg/L and higher compared to non-exposed seedlings (*p* < 0.05) (Supplementary Material Figure S2a).

**Figure 4:**
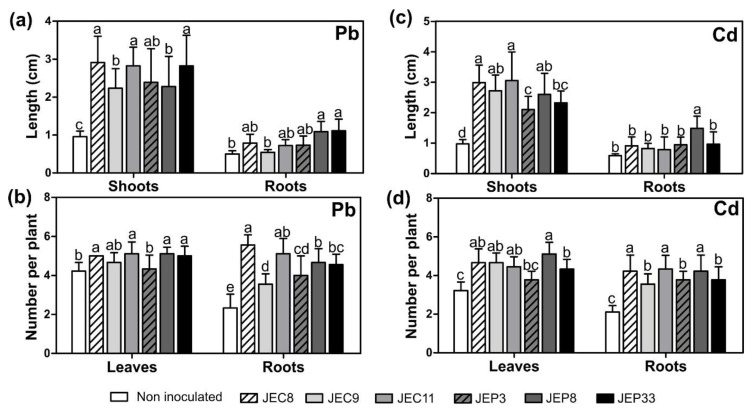
Effect of Pb- and Cd-tolerant isolates in *T. latifolia* seedlings. Pb-exposed *T. latifolia* seedlings (**a**) Length of root and shoots, (**b**) Number of leaves and roots per plant. Cd-exposed *T. latifolia* seedlings, (**c**) Length of root and shoots, (**d**) Number of leaves and roots per plant. Data represent the mean ± SD. Different letters indicate significant differences between treatments (*p* < 0.05).

On the other hand, the roots of *T. latifolia* seedlings were dipped with bacterial strains suspensions and sowed in MS medium plus either 5 mg/L of Pb or 10 mg/L of Cd and incubated for ten days. Results showed that all bacterial strains increased the shoot length of *T. latifolia* seedlings exposed to 5 mg/L of Pb or 10 mg/L of Cd compared with the control plants (Figure 4a,c). Despite that only *P. fluorescens* JEP8 increased root elongation in *T. latifolia* seedlings exposed to Pb or Cd, all bacterial strains increased the number of roots per plant compared with the non-exposed control (Figure 4b,d). 

These results showed that all bacterial strains improve the growth of *T. latifolia* seedlings exposed to 5 mg/L of Pb or 10 mg/L of Cd, suggesting the possible role at the interaction with their host plant in Pb- and Cd-contaminated sites. 

## 3. Discussion

Bacterial isolates were obtained from the roots of *T. latifolia* growing in Pb- and Cd-contaminated environments in higher amounts than the permissible limits set by the WHO [23]. According to bioconcentration factor results, *T. latifolia* roots absorb Cd more efficiently than Pb from the surrounding site. Similar behavior was previously observed in *T. latifolia* growing in a natural wetland to treat municipal wastewater [9]. Furthermore, the results showed that *T. latifolia* roots are colonized by Pb- and Cd-tolerant endophytic bacteria, suggesting that plants select bacteria that improve their growth in Pb- and Cd-contaminated environments. Similarly, Cd-tolerant bacteria have been isolated from the roots of *Sulla coronaria* growing in Cd-contaminated sites [13]. 

Six endophytic bacteria were selected according to their Pb- and Cd-tolerance. They belong to the *Pseudomonas* genus, which includes versatile species with the capacity to adapt to multiple soil conditions, including heavy metals [24]. Previous studies have shown that *Pseudomonas* species are part of endophytic communities in heavy metal hyperaccumulator plants. In *Solanum nigrum* (Cd hyperaccumulator plant), *Pseudomonas* comprises 12.18% of the total endophytic bacteria in its roots [25]. Likewise, endophytic *Pseudomonas* species have been isolated from the root endosphere of *T. angustifolia*, *T. domingensis*, and *Typha* sp. [14,16,26]. These studies indicated that *Pseudomonas* species are part of the microbiome of the *Typhaceae* family and contribute to plant adaptation to heavy-metal-contaminated sites.

According to 16S *rRNA*, *gyrA*, and *rpoD* sequences, JEP3 was identified as *Pseudomonas azotoformans*, JEP8 as *P. fluorescens*, and JEP33 as *P. gessardii*, while JEC8, JEC9, and JEC11 were identified as *P. veronii*. *P. azotoformans* JEP3, *P. fluorescens* JEP8, and *P. gessardii* JEP33 showed higher Pb (MIC > 4.8 mM of Pb) tolerance than *Cupriavidus metallidurans* CH34 (MIC = 1.6 mM of Pb) and *C. metallidurans* BS1 (MIC = 1.7 mM of Pb) [27], while *P. veronii* JEC8, JEC9, and JEC11 exhibited higher Cd (MIC > 5.44 mM of Cd) tolerance than *C. metallidurans* CH34 (MIC = Cd 3.5 mM of Cd) and *C. metallidurans* BS1 (Cd =2.5 mM of Cd) [27]. The high Cd and Pb tolerance of bacterial isolates may be due to their adaptation to high Cd and Pb concentrations in the *T. latifolia* roots. It has previously been shown that high heavy metal concentrations in soils favor the survival of bacteria with multiple resistant mechanisms [28]. However, further studies are necessary to understand the heavy metal resistance mechanisms in bacteria isolated from *T. latifolia* roots. 

Moreover, the six selected bacteria resisted multiple antibiotics (Table 1). Similar results were reported by Dahanayake, et al. [29], who observed that *Aeromonas* isolates resistant to Cd, Pb, and Cr showed resistance to at least five antibiotics. This effect may be because heavy metal contamination induces co-selection of heavy metal and antibiotic resistance genes, which are transferred in bacteria through mobile elements such as transposons and integrons [30,31]. Also, bacterial isolates can grow in LB media plus NaCl, sucrose, and glycerol, suggesting that they could tolerate osmotic stress induced by detrimental environmental conditions such as drought, salinity, and pollutants [32]. 

Plant-growth-promoting mechanisms were determined in six endophytic bacterial strains isolated from *T. latifolia* roots. The results showed that *P. azotoformans* JEP3, *P. fluorescens* JEP8, *P. gessardii* JEP33, *P. veronii* JEC8, *P. veronii* JEC9, and *P. veronii* JEC11 exert PGPR activities, including phosphate solubilization, siderophores production, and IAA synthesis. Except for *P. fluorescens* JEP8, all strains have ACC deaminase activity (Table 2). Plant–bacteria interaction assays demonstrated that these bacterial isolates promote the growth of *T. latifolia* seedlings, showing increased root and shoot length. On the other hand, PGPR activities have been shown to play a beneficial role in their host plants by improving tolerance to heavy metals [33]. Therefore, the bacterial strains were tested in *T. latifolia* seedlings exposed to Pb and Cd at concentrations that decrease their root and shoot elongation. The results showed that the six bacterial isolates *P. azotoformans* JEP3, *P. fluorescens* JEP8, *P. gessardii* JEP33, and *P. veronii* JEC8, JEC9, JEC11 increase shoot length and the root numbers in *T. latifolia* seedlings exposed to Pb or Cd, indicating that bacterial strains improve plant development in presence of these metals. These effects could be attributed to IAA synthesis exerted by bacteria. An extensive review of the effects of IAA-producing bacteria in plants were reported recently [34].

Bacteria isolates in this study have been reported in other studies. *P. azotoformans* ASS1 is an endophytic bacterium, isolated from *Alyssum serpyllifolium*, that resists drought, salinity, antibiotics, extreme temperature, and heavy metals [35]. It increases biomass and leaf water content and improves the Cu, Zn, and Ni accumulation in the roots of wheat *Trifolium arvense* through PGPR activities, including phosphate solubilization, nitrogen fixation, ACC deaminase activity, and siderophore and ammonia production [35]. Moreover, *P. azotoformans* JAW1 accumulates Cd, Cu, and Pb on the cell surface, being an excellent bacterial biosorbent with potential application in bioremediation [36]. Because of its versatility, *P. azotoformans* is used as a biocontrol agent against *Fusarium fujikurio* [37], systemic resistance inducer against *Colletotrichum orbiculare* in *C. sativus* [38], and a good candidate for the preparation of biotechnological concrete due to its capacity to produce calcite [39].

*P. fluorescens* is a versatile PGPR that improves plant growth and yield by increasing antioxidant activities and proline content, and reducing the malanodialdehyde in plants of sunflower (*Helianthus annus* var Hisun-33) [40], rapeseed (*Brassica napus*), and clover (*Trifolium repens*) exposed to Pb. Additionally, *P. fluorescens* has been demonstrated to enhance root Pb accumulation and reduce its translocation to aerial tissues. 

*P. gessardii* produces hydroxamate-type siderophores, solubilizes phosphates [41], reduces Cr (VI) to Cr (III), and degrades naphthalene [42]. Pb-tolerant *P. gessardii* BLP141 has been shown to promote the growth of sunflower *H. annus* var Hisun-33 exposed to Pb (as PbNO_3_) by inducing an antioxidant defense system in plants exposed to it. In addition, *P. gessardii* BLP141 increases the root and shoot length, chlorophyll, and carotenoid content and even increases Pb accumulation in the sunflower roots [40]. It has also been shown that As-resistant bacteria *P. gessardii* and *Brevundimonas intermedia* induce the antioxidant enzymatic response of *Triticum aestivum* exposed to As and increase the shoot growth and dry root biomass of wheat growing in As-polluted soil [41]. Moreover, a consortium of endophytic bacteria including *P. gessardii*, *Aeromonas salmonicida*, *Pseudomonas indoloxydans*, *Bacillus cereus*, and *Rhodococcus* sp. enhance the accumulation of Fe, Mn, Ni, Pb, and Cr in the roots of macrophytes *Brachia mutica*, *T. domingensis*, *Phragmites australis*, and *Leptochala fusca* growing in wetlands to treat polluted river water [17].

Cd-tolerant endophytic *P. veronii* E02 has been demonstrated to exert PGPR abilities, including IAA synthesis, phosphate solubilization, and ACC deaminase activity, which promote the growth of switchgrass (*Panicum virgatum* L.) exposed to Cd [43]. *P. veronii* E02 upregulates the gene encoding the heat shock protein HSP70, which confers Cd tolerance to *P. virgatum* L. [44]. In addition, *P. veronii* E02 induces overexpression of the HMA3A, HMA3B, and HMA3C genes, encoding members of the heavy metal ATPase (HMA) family to decrease Cd accumulation in the roots of switchgrass [44]. Also, *P. veronii* 2E has been demonstrated to produce extracellular polymeric substances acting as biosorbents to retain Cd, Zn, and Cu from aqueous systems [45]. Likewise, this bacterium has been used to reduce Cr (VI) to Cr (III) as a tool to treat industrial wastes and recently as bio-electrochemical systems for monitoring and removal of Cr [46,47]. 

Overall, the six bacterial strains have been previously reported as PGPR in other plant species. Thus, they could exert beneficial effects in their host, *T. latifolia*, by improving its growth and tolerance to Pb and Cd. Further studies are necessary to determine if bacteria improve the phytoextraction of these metals.

## 4. Materials and Methods

### 4.1. Study Site Description and Sample Collection

Samples were collected 2.5 km away from the electrolytic zinc refinery in San Luis Potosi, S. L. P., Mexico, at coordinates 22°09′06.3″ N 101°02′14.3″ W, where Cd contamination has been previously reported [21,48]. Twenty complete plants (15–40 cm long) of *Typha latifolia* and 100 g of surrounding soil were collected in December 2014. The samples were transported to the laboratory in a plastic bag, stored at 4 °C, and used for bacteria isolation and heavy metal analysis. 

### 4.2. Heavy Metal Analysis

Soil samples (10 g) were dried at 70 °C for 24 h, while the plant roots were washed in tap and deionized water to eliminate the adhering soil. Then, the roots were rinsed in EDTA 0.01 M for 10 min to remove heavy metals adsorbed on their surface and dried at 70 °C for 24 h. The dry roots and soil samples were digested according to Rolón-Cárdenas, et al. [49] and analyzed by inductively coupled plasma optical emission spectrometry (ICP-OES; Varian 730-ES, Mulgrave, Australia). Heavy metal concentration was normalized with the dry weight of plants or soil samples and expressed in mg/Kg dry weight (DW). Data were used to calculate the bioconcentration factor (BCF) using the following formula [50]:

(1) $$BCF=\frac{Metal\;roots}{Metal\;soil}$$

where Metal soil and Metal roots (mg/Kg DW) are, respectively, the concentrations of heavy metals in soil and roots of *T. latifolia.*

### 4.3. Isolation of Endophytic Bacteria from T. latifolia Roots

The roots were washed in tap water and surface-sterilized with 75% ethanol and 2% sodium hypochlorite, according to Liu and Xu [51]. Surface disinfection was tested by inoculating 100 µL aliquots from the last rinse in Luria Bertani (LB) plates [14]. The plates were incubated at 28 °C for 72 h, and after incubation, the root samples that did not show bacterial growth were used for endophytic bacteria isolation. 

One gram of surface-sterilized roots was ground in a mortar with 9 mL sterile distilled water, and then tenfold dilutions were prepared. Aliquots (100 µL) of dilutions were inoculated on LB agar at fifth strength (LB 1/5) supplemented with either PbNO_3_ 0.02 mM or CdCl_2_ 0.04 mM (Meyer, Mexico). After incubation at 28 °C for 72 h, bacterial growth was analyzed, and colonies were re-inoculated three times on LB media with Pb or Cd.

### 4.4. Pb and Cd Tolerance of Endophytic Bacteria

Minimum inhibitory concentration (MIC) was determined by the dilution method in 96-well microplates [52]. Serial dilutions 1:2 of Pb and Cd stock solutions were made in LB broth. Plate wells with different concentrations of either Pb (0.3, 0.6, 1.2, 2.4, and 4.8 mM) and Cd (0.34, 0.68, 1.36, 2.72, and 5.44 mM) were inoculated with a bacterial suspension adjusted to 0.5 McFarland standard and incubated 72 h at 28 °C with shaking at 200 rpm. After incubation, bacterial growth was evaluated, and the MIC was established as the minimum concentration of either Pb or Cd that prevents visible bacterial growth [52]. MIC test was repeated three times. The most Pb-tolerant JEP3, JEP8, and JEP33 and most Cd-tolerant JEC8, JEC9, and JEC11 bacterial strains were selected for further analysis. 

### 4.5. Osmotic Tolerance and Antibiotic Susceptibility Test

The most tolerant bacterial strains JEP3, JEP8, JEP33, JEC8, JEC9, and JEC11 were grown in LB broth and incubated at 30 °C overnight. After incubation, bacterial suspensions were prepared in saline solution (NaCl 0.85%) and adjusted to 0.5 McFarland standard. 

For osmotic tolerance, 10 µL of bacterial suspension were inoculated in plates with LB 1/5 supplement with different concentrations of either NaCl, sucrose, or glycerol, ranging from 0.25 to 2.0 M and incubated at 28 °C. The growth was evaluated daily for seven days. 

Antibiotic susceptibility was tested using the disk diffusion method. The bacterial suspension was inoculated on agar Mueller Hinton plates, then a multidisc for gram-negative (Cat. 71080280, Bio-Rad, Hercules, CA, USA) or gram-positive (Cat. 71080180, Bio-Rad, USA) was placed on the surface of independent plates. Plates were incubated for 24 h at 28 °C, and the inhibition zone’s diameter was measured after incubation. Resistance or susceptibility profiles were established according to the manufacturer (Bio-Rad, USA). Multiple antibiotic resistance (MAR) index was calculated according to Patel, et al. [53], using the following formula:

(2) $$MAR\;index=\frac{The\;number\;of\;antibiotics\;the\;isolate\;was\;resistant\;}{Total\;number\;of\;antibiotics\;tested\;}$$

### 4.6. Molecular Identification of Endophytic Bacteria

The most tolerant endophytic bacterial strains JEP3, JEP8, JEP33, JEC8, JEC9, and JEC11 were identified by amplifying and sequencing 16S *rRNA*, *gyrA*, and *rpoD* genes. Bacterial DNA was extracted using the phenol–chloroform method according to Chen and Kuo [54]. PCR amplification was conducted with Platinum PCR SuperMix High Fidelity (Invitrogen, USA), and the primers 27F (5′-AGAGTTTGATCMTGGCTCAG-3′) and 1492R (5′-TACGGYTACCTTGTTACGACTT-3′) for 16S *rRNA* [29]; gyr Fwd (5′-GCAGGTGAGCGATGTAGTCC-3′) and gyr Rev (5′-CCAGGAGATCCTCAACCAGA-3′) for *gyrA* gene; rpoD Fwd (5′-CAGCGACGATGAAGAAGAAG-3′) and rpoD Rev (5′-GGACACAGAGCTGCATGATT-3′) for *rpoD* gene [21]. PCR conditions for 16S *rRNA* were according to Rolón-Cárdenas, et al. [21], while for *gyrA* and *rpoD* were as follows: 95 °C—5 min; 35 cycles of 95 °C—30 s, 55°C—30 s, 72 °C—1 min, and final extension 72 °C—5 min. The PCR product was sequenced at Biotechnology Institute, UNAM (Morelos, Mexico), and the obtained sequence was aligned at GeneBank to determine similarity in the NCBI database.

### 4.7. Determination of PGPR Abilities in Bacteria

#### 4.7.1. Phosphate Solubilization Activity

Bacterial strains JEP3, JEP8, JEP33, JEC8, JEC9, and JEC11 were inoculated in National Botanical Research Institute phosphate (NBRIP) agar with 5% Ca_3_(PO_4_) as a source of insoluble phosphorus (P) and bromocresol purple as a pH indicator. The plates were incubated at 28 °C for 15 days. Phosphate solubilization was determined by a clear halo around the bacteria in NBRIP plates [55]. 

#### 4.7.2. Siderophore Production

The production of siderophores was evaluated using Chrome Azurol S (CAS) agar [31]. Bacterial strains JEP3, JEP8, JEP33, JEC8, JEC9, and JEC11 were inoculated in CAS agar and incubated at 28 °C for 72 h. After incubation, the presence of an orange halo around the colonies was recorded as positive for siderophore production [56]. 

#### 4.7.3. ACC Deaminase Detection

ACC deaminase activity was carried out as described by Penrose and Glick [57]. Bacterial strains JEP3, JEP8, JEP33, JEC8, JEC9, and JEC11 were inoculated in Dworkin and Foster (DF) minimal medium [58], supplemented with ACC (Sigma-Aldrich, St. Louis, MO, USA) 3 mM as the sole source of nitrogen. After incubation at 28 °C for five days, bacteria that grew in DF media were considered positive for ACC deaminase activity. 

#### 4.7.4. Bacterial Auxin Production

IAA and indole-related compounds were determined by the Salkowski colorimetric reaction and GC-MS analysis. Bacterial strains JEP3, JEP8, JEP33, JEC8, JEC9, and JEC11 were inoculated in LB broth supplemented with 1% tryptophan (Trp, Sigma-Aldrich) and incubated at 28 °C for 72 h with shaking at 180 rpm. The supernatant was obtained by centrifugation at 1378.21 g for 15 min. For colorimetric determination, cell-free supernatant (200 µL) was mixed with 800 µL of Salkowski reagent and incubated for 30 min at room temperature in the darkness. Absorbance was determined at 530 nm in a UV–visible spectrophotometer (Evolution TM 201, Thermo Fisher Scientific, Cleveland, OH, USA). The identification of IAA in the bacterial supernatants was determined by GC-MS analysis according to Rolón-Cárdenas, et al. [21]. Ethyl acetate extract was obtained from the supernatant of each bacterial culture and was analyzed by gas chromatography with electron impact mass spectrometry (GC-EIMS, Agilent Technologies, Palo Alto, CA, USA). Quantification of IAA and indole-related compounds were determined using an IAA (Sigma-Aldrich, USA) calibration curve. 

### 4.8. Plant–Bacteria Interaction Assays

#### 4.8.1. Effect of Bacterial Strains in *T. latifolia* Seedlings

Bacterial strains JEP3, JEP8, JEP33, JEC8, JEC9, and JEC11 were grown in LB broth and incubated at 30 °C for 24 h. After incubation, bacterial cells were harvested by centrifugation at 2450 g for 15 min, resuspended in deionized sterile water, and adjusted to OD_600_ = 1.0. 

*T. latifolia* seeds were superficially disinfected with a mixture of 50% sodium hypochlorite solution and 0.02% Triton X-100 for 15 min and rinsed six times with sterile distilled water. Ten disinfected seeds were mixed with 10 mL of each bacterial suspensions and incubated for 2 h at 28 °C with shaking at 180 rpm. After incubation, the seeds were placed on 125 mL glass jars containing sterile water-agar 0.7% (*w*/*v*) and incubated at 28 °C under fluorescent light with a photoperiod 16 h light/8 h dark for 10 days [59]. As a control, non-dipped *T. latifolia* seeds were sowed in water-agar 0.7% (*w*/*v*) and incubated in the same conditions. Finally, the seedlings were recovered to determine the shoot and root length. 

#### 4.8.2. Effect of Bacterial Strains in *T. latifolia* Seedlings Exposed to Either Pb or Cd

First, the effect of Pb and Cd (provided as Pb(NO_3_)_2_ and CdCl_2_, respectively) on *T. latifolia* were determined. Briefly, three ten-day-old *T. latifolia* seedlings were sowed on Murashige and Skoog agar (1% (*w*/*v*) glucose, 3.5 mM of 2-(N-Morpholino) ethanesulfonic acid (MES), 1.6% (*w*/*v*) agar, pH 5.7) supplemented with 0, 5, 10, 20, 30 mg/L of Pb or 0, 10, 20, 30, 40, 50 mg/L of Cd [21]. Plants were incubated at 28 °C under fluorescent light with a photoperiod 16 h light/8 h dark for 10 days. Then, the plants were recovered from the MS agar to determine the root length, and the number of roots and leaves. 

Furthermore, the effect of the bacterial strains JEP3, JEP8, JEP33, JEC8, JEC9, and JEC11 was tested under the conditions described above. Briefly, three ten-day-old disinfected seeds were mixed with 10 mL of each bacterial suspensions and incubated for 2 h at 28 °C with shaking at 180 rpm [59]. After incubation, the seeds were sowed on Murashige and Skoog agar 3.5 mM of 2-(N-Morpholino) ethanesulfonic acid (MES), 1.6% (*w*/*v*) agar, pH 5.7) supplemented with 5 mg/L of Pb or 10 mg/L of Cd [21]. Plants were incubated at 28 °C under fluorescent light with a photoperiod 16 h light/8 h dark for 10 days. Then, the plants were recovered from the agar and determined the root and shoot length, and the number of roots and leaves.

### 4.9. Statistical Analysis

Statistical analysis was performed using the statistical software GraphPad Prism Version 5.01. Analysis of variance (ANOVA) was performed with Tukey’s method to compare the means between treatments [21]. 

## 5. Conclusions

In this study, six PGPR belonging to the *P. fluorescens* group were isolated from *T. latifolia* roots. Plant–bacteria interaction assays demonstrated that bacterial isolates promote the growth of *T. latifolia* seedlings in the presence and absence of either Pb or Cd. To our knowledge, this is the first report that describes the presence of cultivable endophytic bacteria *P. azotoformans*, *P. gessardii*, *P. fluorescens*, and *P. veronii* inside *T. latifolia* roots grown in Pb- and Cd-contaminated environments. Therefore, this study extends the knowledge about endophytic bacteria, which could contribute to *T. latifolia* adaptation to heavy metal polluted sites.

## Figures and Tables

**Table 1:** Molecular identification of heavy metal resistant-endophytic bacteria.

Strain	Identity	Gene Bank Accession Number		
		16S *rRNA*	*gyrA*	*rpoD*
JEP3	*Pseudomonas azotoformans*	MT280006	MW924893	MW924899
JEP8	*Pseudomonas florescens*	MT280007	MW924894	MW924900
JEP33	*Pseudomonas gessardii*	MT280008	MW924895	MW924901
JEC8	*Pseudomonas veronii*.	MT280009	MW924890	MW924896
JEC9	*Pseudomonas veronii*.	MT280010	MW924891	MW924897
JEC11	*Pseudomonas veronii*	MT280011	MW924892	MW924898

Gene bank accession number in the National Center for Biotechnology Information (NCBI) database.

**Table 2:** Antibiotic test of endophytic bacteria from *T. latifolia* roots.

Class	Antibiotic	Content (µg)	Strain					
			JEP3	JEP8	JEP33	JEC8	JEC9	JEC11
Penicillins	Penicillin (PEN)	6 (10 IU)	S	S	S	S	S	S
	Ampicillin (AMP)	10	S	S	S	S	S	S
	Carbenicillin (CAR)	100	S	S	S	S	S	S
	Dicloxacillin (DCX)	1	S	S	S	S	S	S
Tetracyclines	Tetracycline (TET)	30	R	R	R	R	R	R
Cephalosporins	Cephalothin (CEF)	30	S	S	S	S	S	S
	Ceftriaxone (CRO)	30	I	R	S	S	S	R
	Cefotaxime (CTX)	30	S	S	S	S	S	S
	Ceftazidime (CAZ)	30	S	S	S	S	S	S
	Cefuroxime (CXM)	30	S	S	S	S	S	S
Quinolones	Pefloxacin (PEF)	5	R	R	R	R	R	R
Macrolides	Erythromycin (ERY)	15	S	S	S	S	S	S
Aminoglycoside	Amikacin (AMK)	30	R	R	R	R	R	R
	Gentamicin (GEN)	10	R	R	R	R	R	R
	Netilmicin (NET)	30	R	R	R	R	R	R
Phenicols	Chloramphenicol (CHL)	30	I	S	S	R	S	S
Nitrofurans	Nitrofurantoin (NIT)	300	S	S	S	S	S	S
Diaminopyrimidine/Sulfonamides	Trimethoprim/Sulfamethoxazole (SXT)	25	S	S	S	S	S	S
MAR index			0.28	0.33	0.28	0.33	0.28	0.33

(S) Susceptible; (I) Intermediate; (R) Resistant; (MAR) Multiple antibiotic resistance. JEP3 = *P. azotoformans*; JEP8 = *P. fluorescens*; JEP33 = *P. gessardii*; JEC8, JEC9, JEC11 = *P. veronii.* Data in parentheses are antibiotics abbreviation.

**Table 3:** Characteristics of endophytic bacteria from *T. latifolia* roots.

Strain	Siderophore Production	ACC Activity	Solubilization Ca(PO_3_)_2_	Indole Compounds Production (µg/mL)	
				Salkowski	IAA
JEP3	+	+	+	23.2 ± 1.3 ^ab^	0.079 ± 0.001 ^bc^
JEP8	+	−	+	17.5 ± 1.5 ^b^	0.094 ± 0.009 ^b^
JEP33	+	+	+	14.7± 0.5 ^bc^	0.069 ± 0.0004 ^c^
JEC8	+	+	+	31.9 ± 7.1 ^a^	0.080 ± 0.002 ^bc^
JEC9	+	+	+	6.3 ± 1.0 ^c^	0.115 ± 0.013 ^a^
JEC11	+	+	+	8.4 ± 2.0 ^c^	0.080 ± 0.003 ^bc^

(+) Positive; (−) Negative. Data represent the mean ± SD. Different superscripts indicate significant differences in production of indole compounds (Salkowski reaction) and IAA (GC-MS quantitation) between strains (*p* < 0.05).

## Data Availability

The data supporting this study’s findings are available from the corresponding author, A.H.-M., upon reasonable request.

## Supplementary Materials

The following supporting information can be downloaded at: https://www.mdpi.com/article/10.3390/plants12030498/s1, Figure S1: Effect of Pb in *T. latifolia* plants; Figure S2: Effect of Cd in *T. latifolia* plants.
