# Supplementary material for: Characterization of Endophytic Bacteria Isolated from Typha latifolia and Their Effect in Plants Exposed to Either Pb or Cd
Source: Plants (Basel). 2023 Jan 21;12(3):498. doi: 10.3390/plants12030498 (PMC9920544; doi:10.3390/plants12030498)
Supplement: Supplementary file 1 [file plants-12-00498-s001.zip › plants-1789819-supplementary.pdf]

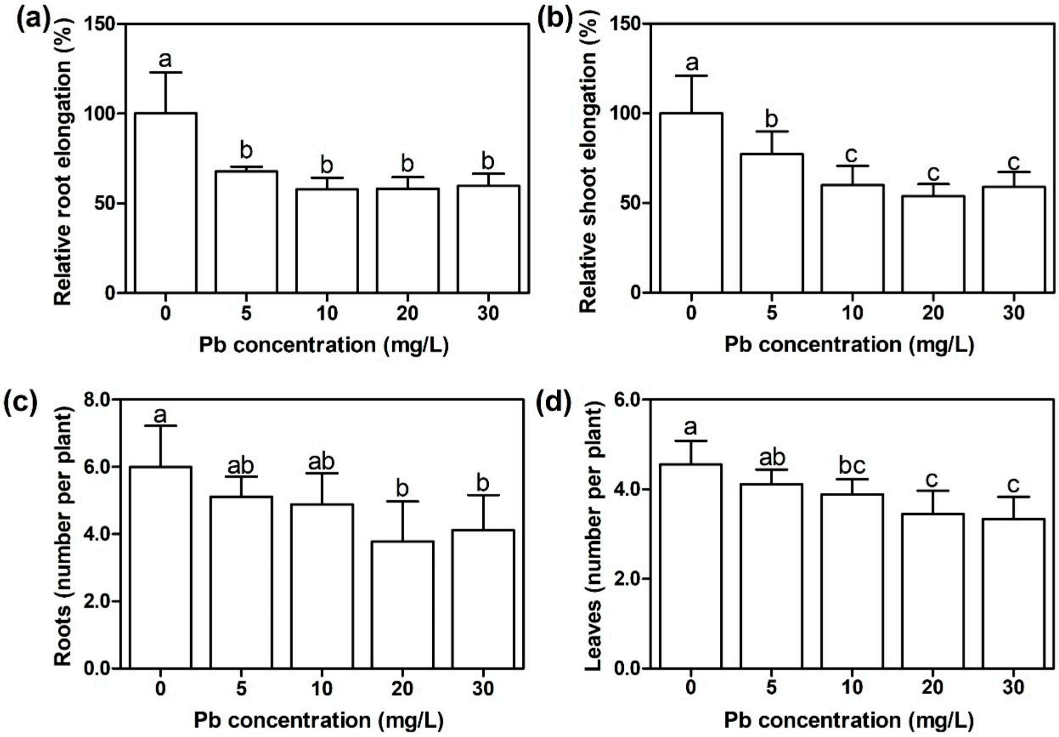

Figure S1. Effect of Pb in *T. latifolia* plants. The values represent the mean ± SD (n = 9). Different letters indicate significant differences between treatments.

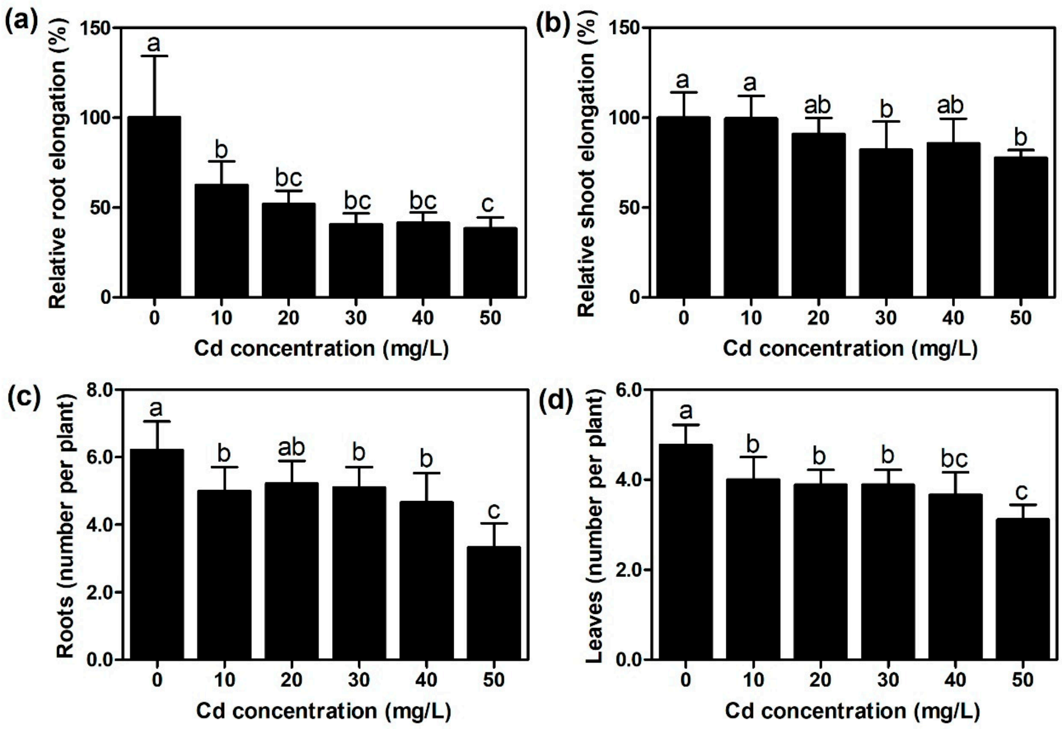

Figure S2. Effect of Cd in *T. latifolia* plants. The values represent the mean ± SD (n = 9). Different letters indicate significant differences between treatments.
